# Molecular targets and mechanisms of cortex mori for lung cancer treatment: a network pharmacology study, molecular docking and *in vitro* and *in vivo* experimental validation

**DOI:** 10.3389/fonc.2025.1587856

**Published:** 2025-05-29

**Authors:** Ying-Ying Shao, Qiu-Hong Yang, Shu-Fen He, Han-Bin Zhang, Wei-Chao Han, Bao-Cheng Xie, Rui-Rong He, Wen-Xin Hong

**Affiliations:** ^1^ Department of Health, Dongguan Maternal and Child Health Care Hospital, Dongguan, China; ^2^ Department of Pharmacy, The Tenth Affiliated Hospital of Southern Medical University (Dongguan People’s Hospital), Dongguan, China; ^3^ School of Pharmacy, Zunyi Medical University, Zunyi, China

**Keywords:** lung cancer, cortex mori, network pharmacology, mechanism of action, target, pathway

## Abstract

**Introduction:**

The cortex mori comes from the white endothelium of the young root of Morus alba L., and its medical value was first described in Shen Nong Ben Cao Jing (Classic on Materia Medical of Shennong). It was originally intended to purge lung, relieve asthma and reduce swelling. More and more studies reported that its pharmacological effects include analgesic, anti-inflammatory, antitussive, antiasthmatic, hypoglycemic, hypolipidemic and anti-diabetic peripheral neuropathy. Accumulating clinical evidences exhibited that it can treat asthma, pneumonia and lung cancer. However, a comprehensive mechanism of cortex mori in the treatment of lung cancer needs to be further elucidated.To investigate the effect of cortex mori and its active components against lung cancer and explore its action and mechanism through network pharmacological analysis combined with biological experiments *in vitro* and *In vivo*.

**Methods:**

GeneCards database was searched for the disease targets of lung cancer, and a Chinese medicine database, Traditional Chinese Medicine Systems Pharmacology (TCMSP), was used to screen cortex mori for its active components and targets. Targets related to lung cancer and action targets related to cortex mori were crossed. Protein-protein interactions (PPI) and gene ontologies (GO) and Kyoto Encyclopedia of Genes and Genomes (KEGG) were analyzed for intersection genes. In order to determine whether cortex mori affects lung cancer, MTS, wound healing, Western-blot, Hoechst assay, apoptosis assay and animal experiments were performed.

**Results:**

32 active ingredients and 434 targets of Chinese medicine cortex mori were obtained. Totally 2,3107 lung cancer related targets were collected, and 163 Chinese medicine-disease targets were derived from the intersection. The regulatory network of Chinese medicine-active ingredient-disease-targets showed that cortex mori acted on 163 disease targets of lung cancer mainly by cyclomolorusin, kuwanon D and Moracin A, etc. The core genes involving cortex mori treating lung cancer might consist of JUN, AKT1, etc. The core targets involved 162 biological processes, mainly including nuclear receptor activity, ligand-actived transcription factor activity, etc. The core study targeted 160 pathways, including AGE-RAGE signaling pathways associated with diabetes complications, fluid stress and atherosclerosis. Biologic cytological experiments showed that the effective active component cyclomorusin inhibited proliferation, inhibited migration and induced apoptosis of lung cancer through AKT-PI3K pathway. *In vivo* antitumor assay demonstrated that cyclomolorusin suppressed the tumor growth in mice.

**Discussion:**

Cortex mori acts on AKT and other related disease targets of lung cancer cells through effective components such as cyclomolorusin, and plays a role in the treatment of lung cancer by inhibiting the signaling pathway associated with lung cancer occurrence and development.

## Introduction

As one of the most common malignant tumors worldwide, lung cancer has one of the highest mortality rates. Early lung cancer patients usually do not show obvious symptoms, and many patients are diagnosed in the late stages with distant metastases, so they lose the opportunity of surgical resection or radiotherapy (1). Chemotherapy is often used in clinical treatment, while it often combined with the toxic side effects. The effective improvement of chemotherapy and the reduction of harmful side effects of drugs is an urgent need today (2).

It has been thousands of years since Traditional Chinese Medicine (TCM) developed in China. It has mild effects and can also exert certain inhibitory effects on cancer cells, so it has become the focus of research and development of cancer chemotherapy drugs. Cortex mori is the dry root bark of Morus alba L. It can purge lung, relieve asthma and reduce swelling (3). Cyclomorusin is a kind of medicinal components isolated from cortex mori. Invasion and wound healing experiments, and the results showed that methylene chloride extract of mulberry white skin inhibited the migration and invasion of tP4 and NSCLC cells in H1299, H460 and A549 in a concentration-dependent manner (4). Oligo-chitosan from cortex mori inhibited tumor growth and prolonged the survival time of mice with tumors, and its effect may be related to improve the immunity (5). Cortex mori has been proved to have significant antitumor effect (6, 7). The above studies preliminarily confirmed that various extracts of cortex mori have certain anti-tumor effects. A variety of components from cortex mori have been confirmed to have good inhibitory effects on cell proliferation of NCI-H460 lung cancer cells, but the active molecular and its mechanism need to be further studied (8). Therefore, the mechanism of cortex mori in lung cancer is worth further to be investigated.

Based on systems biology and network pharmacology, network pharmacology aims to investigate the active components of traditional Chinese medicine in disease and related pathways and biological function. In addition to improving therapeutic effects, reducing side effects, and increasing the success rate of clinical trials. Network pharmacology is a new main method and direction in traditional Chinese medicine research (9–12). Therefore, an investigation of the mechanism of action of cortex mori in lung cancer treatment was performed using network pharmacology technology, in order to offer a new method and direction for the development of traditional Chinese medicine of cortex mori.

**Figure 1:**
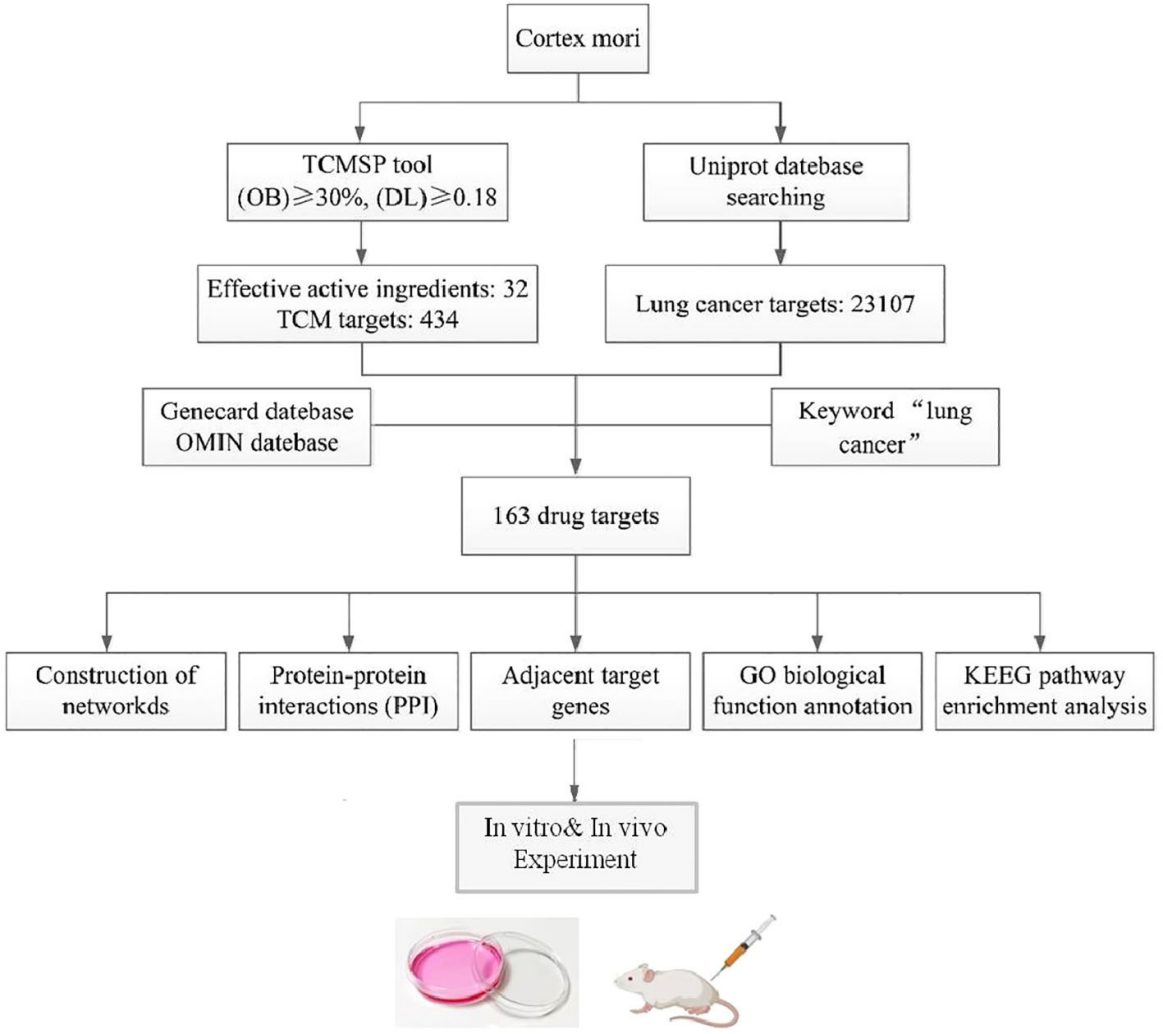
The flowchart of the network pharmacology research and experiments of cortex mori against lung cancer.

For many years, there have been reports on the anticancer effects of cortex mori, but its major active ingredient and associated molecular mechanism remain unknown. Therefore, an active component of cortex mori was screened using network pharmacology in this study, and the protein interaction network of cortex mori provided a reference for further anticancer research by analyzing its biological processes and signaling pathways. The workflow of network pharmacological analysis and experiments is illustrated in **Figure 1**.

## Materials and methods

### Cortex mori active ingredients screened

The Traditional Chinese Medicine Systems Pharmacology Database (TCMSP http://lsp.nwu.edu.cn/) and Analysis Platform was searched using the keywords ‘cortex mori’ and related literature was reviewed for all chemical ingredients. The related compounds were identified through a screening of active compounds with oral bioavailability (OB)>30% and drug likeness (DL)>0.18 (13, 14). OB is primarily a measure of how much the drug is absorbed into the body circulation, and the DL value is primarily used to determine how similar a compound is to known drugs, and these two sources provide a valuable reference for active ingredient analysis (15, 16). To improve accuracy and uniformity, target information and gene name standardization are compared in the UniProt database (https://www.uniprot.org/help/uniprotkb).

### Acquisition of target sites of cortex mori

Traditional Chinese Medicine Systems Pharmacology Database (TCMSP, http://tcmspw.com/tcmsp.php) and Analysis Platform was searched for the keywords ‘cortex mori’ to find all effect targets. For target gene selection, chose ‘Related Targets’ from the results obtained with Perl-5.32.0.1 software (https://www.perl.org/get.html).

### Acquisition of disease-related genes

On the basis of the Online Mendelian Inheritance in Man (OMIM, https://www.omim.org) and Human Gene Database (GeneCards, https://www.genecaed.org), we identified a number of gene targets linked to lung cancer. A Venn diagram was drawn using the information obtained from UniProt (https://www.uniprot.org/help/uniprotkb), including their names, functions, symbols, and gene IDs. A comprehensive list of known gene targets was provided, facilitating further research and analysis of ‘drug-disease’.

### Construction of networks

Screened pharmaceutically active ingredients were introduced into the common target gene for ‘drug-disease’ interactions, and with Cytoscape 3.7.2 (http://www.cytoscape.org/), an interface was created for the visual composition network between active ingredient and cancer target. The nodes on the network diagram represent corti mori and gene proteins, and the edges represent the relationships between active ingredients and targets of action. In addition, the number of lines connecting target genes in a network determines the significance of node-to-node interactions.

### Protein-protein interaction network construction

The ‘drug-disease’ target was delivered into the STRING biological database (https://string-db.org/) and at Organism, sorted the parameter ‘homo sapiens’ for studying target protein interactions, saved the result file. The obtained files were entered into R×64 4.0.2 software for calculation, and the bar graph of the core targets of the protein interaction network was generated. PPI diagrams showed gene structures, proteins, and their relationships, with solid circles representing genes, and different colored lines connecting the circles. Lines represent biological processes involving proteins, including gene expression regulation, signal transduction, and cell migration.

### GO and KEGG enrichment analysis

A version of R 4.0.2 was installed with the packages ‘colorspace’, ‘string’ and ‘ggplot2’ and the analysis of GO and Kyoto Encyclopedia of Genes and Genomes (KEGG) enrichment was performed using a bioconductor package that includes ‘DOSE’, ‘clusterProfiler’, and ‘enrichplot’. Among them, it was considered significant when the P-value was less than 0.05, which indicated a strong correlation between cancer and the target protein. Biological functions of target proteins or related pathways may be regulated by cortex mori to induce anti-lung cancer effects. A bar graph was produced based on the analysis of the first 20 enrichment results.

### Molecular docking stimulation

A search of the PDB database (https://www.rcsb.org) was conducted to find the target genes involved in the first 10 pathways of the KEGG enrichment results, of which by X crystal diffraction, 3D protein conformations with a crystal resolution lower than three were determined. Mol2 format files of the key active ingredients in GGQL were downloaded from the TCMSP platform. Proteins were processed using AutoDock Tools 1.5.6, which separated proteins, added nonpolar hydrogen, calculated Gasteiger charges, and assigned AD4 types, and turned all flexible bonds of small molecule ligands into rotatable bonds. Based on the original coordinates of the ligand and target protein, the docking box has been adjusted to include the target protein’s structure. We selected the genetic algorithm for rigid docking and set the medium to the maximum number of evaluations when we docked the receptor protein rigidly. Autogrid4 and Autodock4 were used to obtain docking results, where binding energies were revealed. A partial docking diagram was created using PyMol software.

### Cell culture and reagents

Jiajia Cui (Department of Clinical Pharmacology, Xiangya Hospital, Central South University, Changsha, China) kindly provided NCI-H1299 and A549 lung cancer cell lines. Cells were grown in 1640 medium containing 10% fetal bovine serum (BI) at 37°C in an incubator with 5% CO2. Cyclomorusin was purchased from Chengdu must bio-technology company (Chengdu, China), its quality was conducted by Chengdu must bio-technology company and the quality of cyclomorusin meet the reporting standards (**Supplementary Table S1**). Shanghai Yuanye Bio-technology Company (Shanghai, China) supplied cortex mori and cis-platinum; Sigma-Aldrich (St. Louis, MO, USA) supplied dimethyl sulfoxide (DMSO). We dissolved cyclomorusin, cortex mori, and cis-platinum in DMSO. The DMSO concentration in the media was limited to 0.1% (v/v). DMSO treated cells were used as vehicle controls only.

### Cell proliferation assay

Five thousand cells were plated into 96-well plates at a density of 5×10^3^, and treated for 24 and 48 hours with various concentrations of cyclomorusin, cortex mori and platinum, or 0.1% DMSO (isotype control). By using the MTS assay (Promega) based on the manufacturer’s instructions, cyclomorusin, cortex mori, and cis-platinum were evaluated on proliferation of A549 and NCI-H1299 cells. A solvent (only) used to dissolve cyclomorusin, cortex mori, and cis-platinum did not affect lung cancer cells’ viability.

### Hoechst 33342 staining

Lung cancer cells were cultured for 12 hours in 35 mm corning dishes. After 24 hours of co-incubation with cyclomorusin, the culture medium was removed and fixed with 4% paraformaldehyde for 10 minutes. Lung cancer cells were washed with PBS three times, followed by 10 minutes of staining with Hoechst 33342 (10 mg/mL). Cells were washed with PBS and photographed by confocal microscopy.

### Apoptosis assays

Flow cytometry analysis was performed using Annexin V-FITC Apoptosis Detection Kit (KeyGen bio-technology company, Jiangsu, China) following treatment with cyclomorusin and cis-platinum for 48 hours.

### Detection of cell cycle

Lung cancer cells were treated with cyclomorusin in 6-well plates for 24 h. After incubation, cells were collected and fixed with cold 70% ethanol at -20°C overnight. The cells were centrifuged (15 minutes at 800 g) and we washed the cells twice with cold PBS, then put back in 500-mL PBS containing PI (50 g/mL) and DNase-free RNase (100 g/mL), analyzed using flow cytometry.

### Wound healing assay

A six-well plate was cultured with lung cancer cells (A549 and NCI-H1299). The supernatant was absorbed from the cells after they had reached nearly 100% confluence and the cells were scraped with a pipette tip sized for 10 liters. In the following step, the cells were washed in PBS to remove detached cells, and medium was added with cyclomorusin and cis-platinum. In order to determine the extent of metastasis of cells, a microscope was used at 0, 24 and 48 hours after the cells had been spread. During remodeling, the distance across the injured region diminished, normalized to the control at 0 h, and expressed as the extent of outgrowth. The solvent used to dissolve cyclomorusin and cis-platinum, lung cancer cells exhibited no differences in their migration properties.

### Western blot analysis

The lysis buffer for the Radio Immunoprecipitation Assay (RIPA) was used to extract the cells at ice-cold temperatures (Beyotime, P0013B). We measured the protein concentration in lysates of tissues and cells using a BCA kit (Beyotime, P0010S). By electrophoresis on SDS-PAGE, 10L of proteins were separated, transferred to PVDF membranes and blocked for two hours with 5% skim milk powder, and then incubated with various primary antibodies at 4°C overnight. TBST was used three times, each for 10 minutes, to wash primary antibodies before adding secondary antibodies. After shaking at room temperature for 2 hours, the secondary antibodies were removed and washed with TBST three times for 10 minutes each time, using the ECL system (BIO-RAD, Hercules, CA, USA) to show the protein bands. The primary antibodies for GADPH, p-PI3K (p85), PI3K (p85), p-AKT, AKT, p-mTOR, mTOR, Bcl-2, Bax, N-Cadherin and Vementin were purchased from Cell Signaling Technology.

### Colony formation assay

To assay the effects of cyclomorusin on colony formation of lung cancer cells, 400 viable lung cancer cells (NCI-H1299 andA549) were seeded in six-well plates in triplicate for 24 h, then continually maintained in complete medium with or without a series doses of cyclomorusin for 10 days. Foci were fixed with 4% polyoxymethylene and stained with 0.1% crystal violet (Beyotime, C0121, Shanghai, China). The stained foci were washed by Phosphate Buffered Saline (PBS) three times and then detected by microscope.

### The effects on zebrafish embryo development

Embryos of zebrafish were obtained from Shanghai FishBio Co., Ltd. All animal experiments were approved by Dongguan People’s Hospital’s Ethics Committee. We conducted the treatments in 24-well plates and each well contained 15 normal zebrafish embryos with 1 mL embryo medium. We set up blank control group, 1% DMSO and five gradient concentrations of cyclomorusin groups. After 24 h, 48 h, 72 h, 96 h treatment, we used stereomicroscopy to monitor embryo development. The hatching rate and malformation rate at different time points were calculated and analysed (The hatching rate = hatching embryo/total number of embryos * 100%; The malformation rate = the number of the malformation embryo/the number of the embryos alive * 100%).

### *In Vivo* anti-tumor assays

We purchased several female nude mice (4 weeks old) from Southern Medical University’s Experimental Animal Centre (Guangzhou, China). All procedures were approved by Dongguan People’s Hospital’s Ethics Committee. All mice were randomly divided into three groups with eight mice per group after seven days of adaptive raising. Mice were injected subcutaneously with NCI-H1299 (5×10^6^). We randomly divided mice into three groups (n=5) after the tumors reached an average size of 75–100 mm3 and then treated with solvent control, cyclomorusin (15 mg/kg, 30 mg/kg). We observed mice daily and measure the tumor’s size and weight the two-day. We calculated tumor volumes based on the following formula: tumor volume (V) = (tumor length) × (tumor width)^2^/2. V/V0 is calculated as the relative tumor volumes, and V0 is the initial volume of the tumor. In the end of the experiment (15 days), we killed the mice, and isolated the tumor, heart, spleen, liver, lung, and kidney. Accordingly, tumor rate = [1 – (*V*− *V0*)/(*V*′ − *V0*′)] × 100%, *V* and *V*′are the tumor volumes of treatment and solvent control groups, respectively. In the treatment group, *V0* is the initial tumor volume, while in the solvent control group, *V0*′is the initial tumor volume.

### Statistical analysis

Except in special circumstances, all data (n=3) are presented as mean ± standard deviation. Student’s test and one-way analysis of variance were used as statistical measures. P < 0.05 was considered statistically significant.

## Results

### Screening of active ingredients and related targets

Under the conditions of OB > 30% and DL > 0.18, the TCMSP and UniProt databases were searched and 32 active components and 434 related targets were identified. The active ingredients include cyclomorusin, kuwanon D, Moracin A and moracin O etc. (**Table 1**). There had been numerous clinical trials demonstrating the potency of cortex mori’s active ingredients in preventing lung cancer *in vivo* and *in vitro*, but the mechanism needed to be explored further. The drug-likeness value of cyclomorusin was the top 1 among the major active ingredients in cortex mori. In our study, the mechanism of cyclomorusin was chosen for further investigation.

**Table 1:** The active ingredients of cortex mori.

Active ingredient No.	Active ingredient name	Oral bioavailability (OB)	Drug-likeness (DL)
MOL012681	Dimethyl (methylenedi-4,1-phenylene) biscarbamate	50.84	0.26
MOL012686	7-methoxy-5,4’-dihydroxyflavanonol	51.72	0.26
MOL012689	Cyclomorusin	36.79	0.87
MOL012692	Kuwanon D	31.09	0.8
MOL012714	Moracin A	64.39	0.23
MOL012717	Moracin M-6,3’-di-O-β-D-glucopyranoside	37.81	0.74
MOL012719	Moracin O	62.33	0.44
MOL012726	Mulberrofuran G	92.19	0.24
MOL012735	Mulberroside C_qt	71.39	0.46
MOL012743	Resveratrol-3,4’-di-O-β-D-glucopyranoside	35.08	0.76
MOL012749	Sanggenone B	115.44	0.3
MOL012753	Sanggenone F	62.42	0.54
MOL012755	Sanggenone H	37.5	0.53
MOL012760	Sanggenone M	68.29	0.85
MOL001474	Sanguinarine	37.81	0.86
MOL000211	Mairin	55.38	0.78
MOL000358	Beta-sitosterol	36.91	0.75
MOL003758	Iristectorigenin (9CI)	71.55	0.34
MOL003856	Moracin B	55.85	0.23
MOL003857	Moracin C	82.13	0.29
MOL003858	Moracin D	60.93	0.38
MOL003860	Moracin F	53.81	0.23
MOL004912	Glabrone	52.51	0.5
MOL000098	quercetin	46.43	0.28
MOL001004	pelargonidin	37.99	0.21
MOL012800	3,5,7-trihydroxy-2-(3-hydroxyphenyl) chromone	59.71	0.24
MOL002514	Sexangularetin	62.86	0.3
MOL000422	kaempferol	41.88	0.24
MOL005043	campest-5-en-3beta-ol	37.58	0.71
MOL000554	gallic acid-3-O-(6’-O-galloyl)-glucoside	30.25	0.67
MOL009653	Cycloeucalenol	39.73	0.79
MOL000098	quercetin	46.43	0.28

### Prediction of anti-lung cancer targets of active ingredients and drug-active ingredient-target gene network construction and analysis

**Figure 2:**
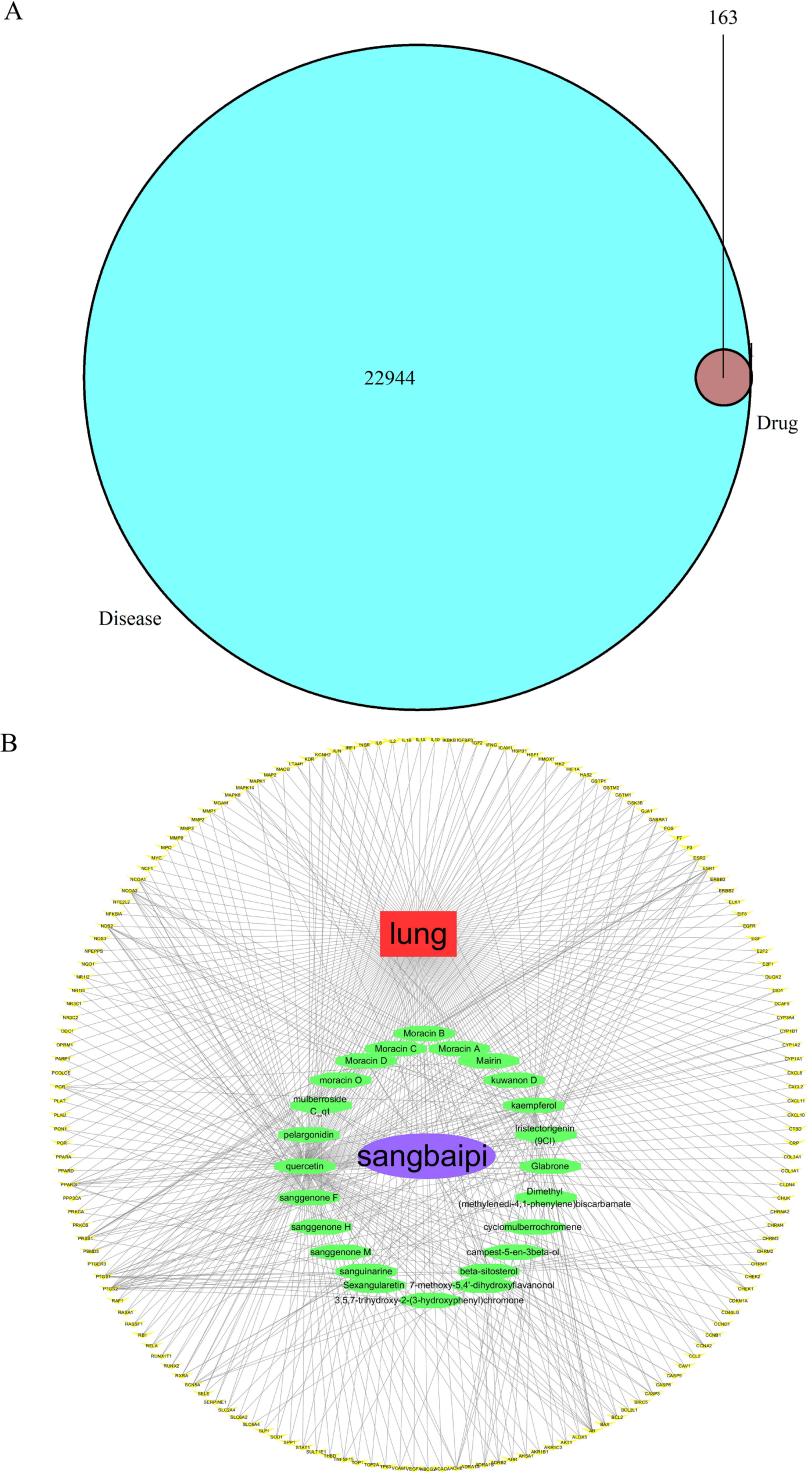
Prediction of anti-lung cancer targets of active ingredients and drug-active ingredient-target gene network construction and analysis. **(A)** Venn diagram of TCM-disease target. 22944 target genes were identified and 163 drug-disease targets were identified according to intersections. **(B)** TCM-ingredients-disease-target regulatory network. Drug-component target network of active ingredients of cortex mori.

Based on Gene Cards and OMIM databases, 22944 target genes were identified, and 163 drug-disease targets were identified according to intersections (**Figure 2A**). A Venn diagram was a diagram used to show areas where collections of elements overlap. In the Venn diagram, the large circle represented the relevant target of the disease, the small circle represented the target of the drug, and the intersection part was the target of the drug on the disease. A visualization of the active ingredient-cancer target network was constructed using Cytoscape 3.7.2 software, which included 32 effective active ingredients, cancer, and 163 ‘drug-disease’ key target genes (**Figure 2B**). In the constructed regulatory network, red represented lung cancer, purple oval represented the TCM cortex mori, green oval represented the active component of cortex mori, and yellow V-shape represented the TCM-disease target. Based on the analysis of the network, each active ingredient targets at least one gene, and each gene is regulated by at least two active ingredients, and a multi-component and multi-target action model for cortex mori is presented.

### Analysis of target protein interaction network

**Figure 3:**
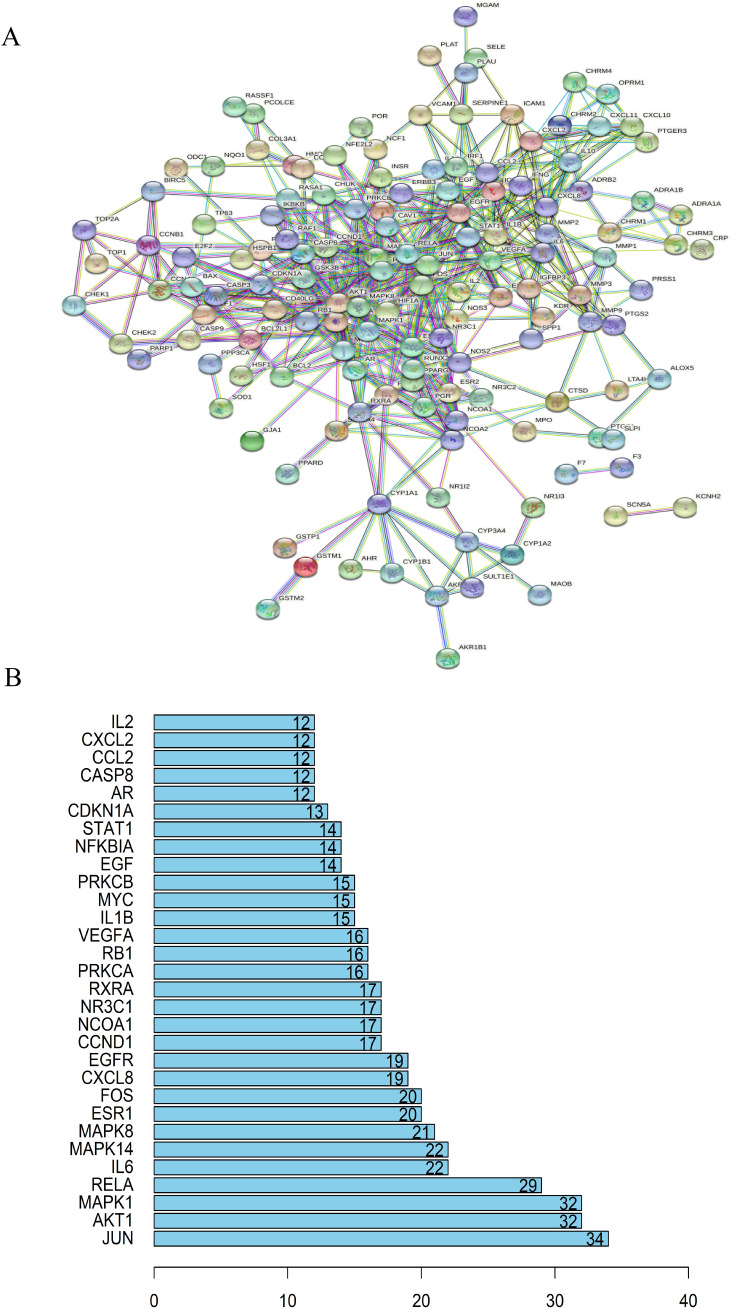
Analysis of target protein interaction network. **(A)** TCM-disease target protein interaction network. Anti-cancer protein target interaction network (PPI). **(B)** A bar chart of TCM-core disease targets. MAPK1, AKT1 and JUN were the three proteins with more than 30 connectivity degrees and may be key targets for anticancer therapies.

An interaction network between target proteins was constructed from the STRING database, and lung cancer target genes and active components of the cortex mori were imported. The selected species was ‘Homo sapiens’, and the combined score exceeded 0.4. **Figure 3A** shows the final protein-protein interaction network threshold. Each dot in the PPI network represented a key target gene, with its center displaying its protein structure. There were 163 solid circles of different colors in the network. The links between the target genes of the PPI network represented different aspects of gene interaction, including protein homology, genes co-expression, genes co-evolution, and inter-genetic relationships. Based on statistical analyses of each target gene, we identified the 30 proteins with the highest number of adjacent genes (**Figure 3B**). There were three proteins with more than 30 connectivity degrees: MAPK1, AKT1 and JUN. When genes are located closely together in a network, their roles become more prominent, and these genes may be key targets for anticancer therapies.

### Enrichment of related targets from GO and KEGG

**Figure 4:**
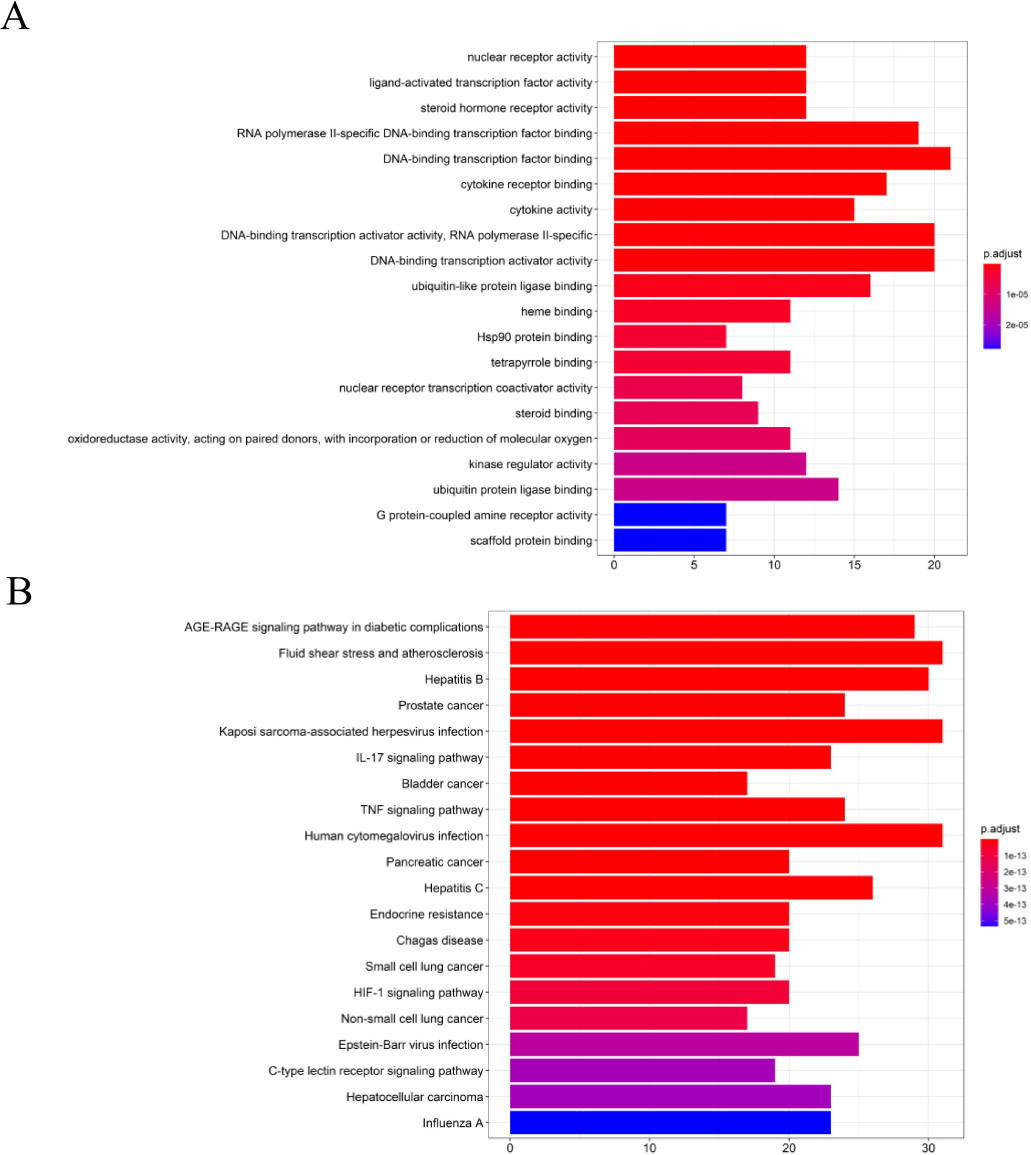
Enrichment analysis of the core targets from PPI analysis. **(A)** Histogram of GO functional enrichment analysis of anti-cancer targets gene from active ingredients of cortex mori. **(B)** Enrichment analysis histogram of KEGG pathway of anti-cancer targets gene from active ingredients of cortex mori.

**Figure 5:**
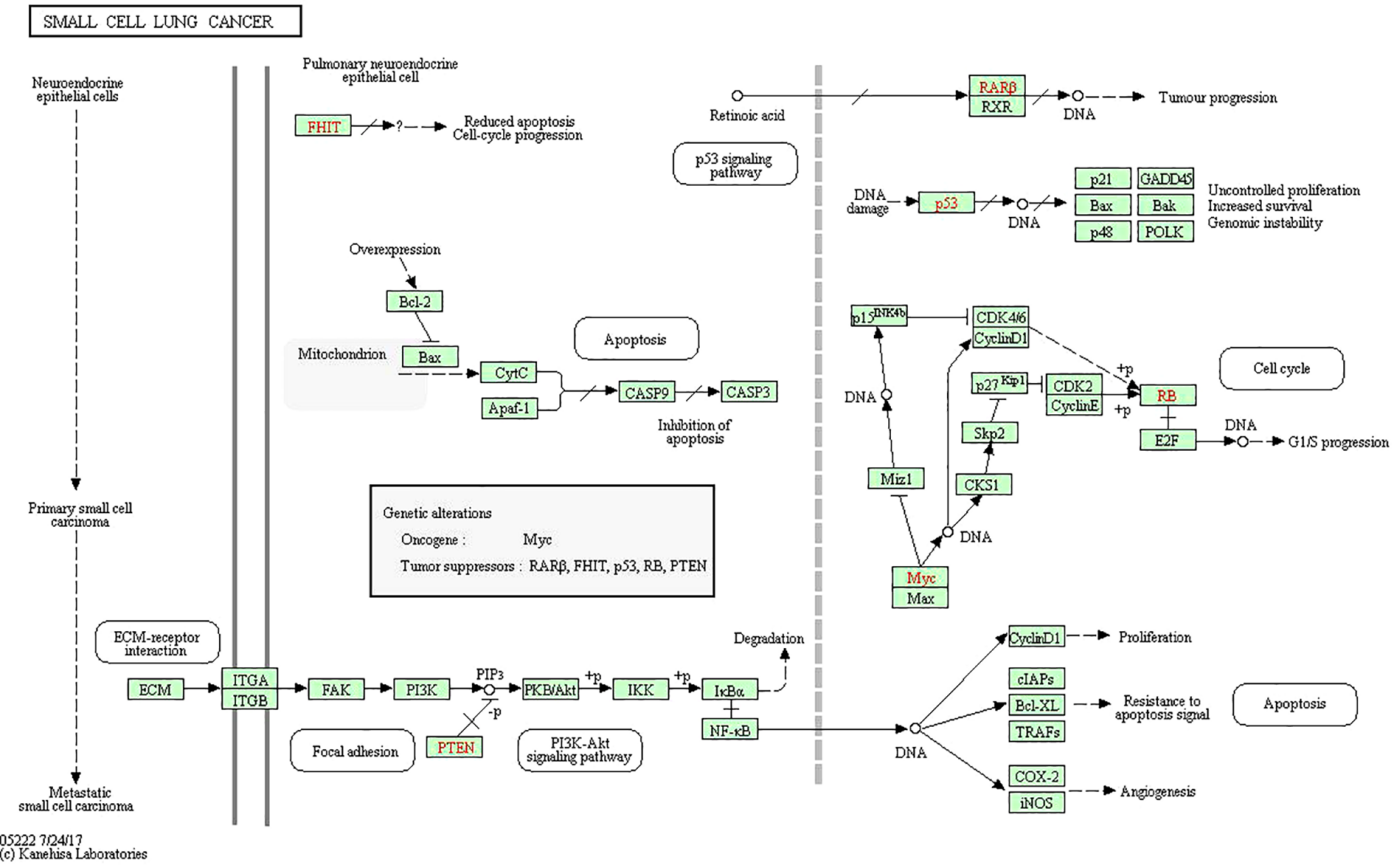
Small cell lung cancer pathway including PI3K-Akt signaling pathway and p53 signaling pathway.

**Figure 6:**
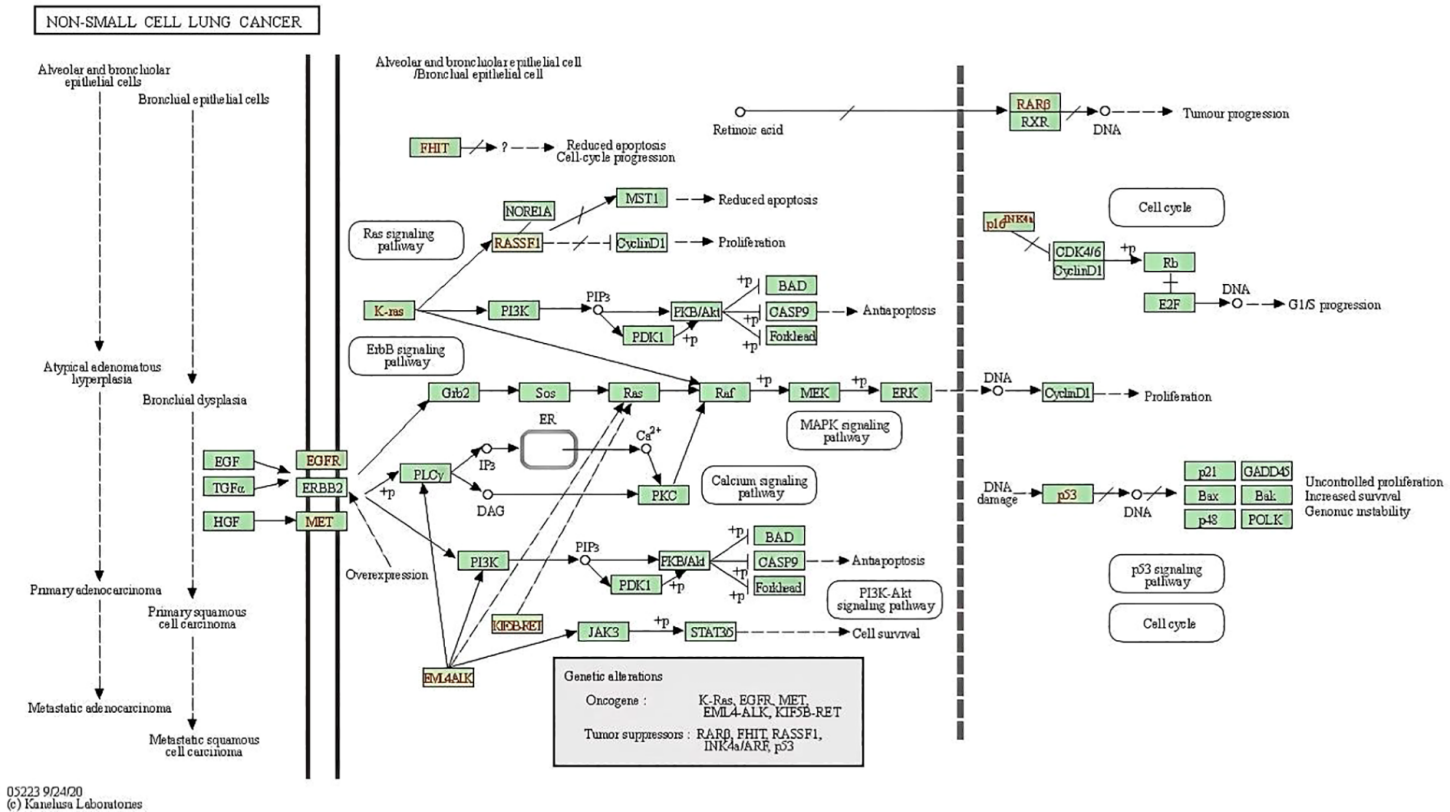
Non-small cell lung cancer pathway including PI3K-Akt signaling pathway and p53 signaling pathway.

To analyze 32 targets genes at the intersection of the active ingredient in the drug and the cancer, bioinformatics tools from Bioconductor were used. To filter the data, we used P value = 0.05 and Q value = 0.05, and we enriched and analyzed the GO biological function and KEGG signaling pathways using R statistical software. A total of 162 biological processes were identified through the enrichment of GO biological functions. In order to analyze the functional terms, we selected the top 20 enriched functional terms. Generally, a higher P value indicates a greater enrichment in terms of biological significance. As a result of the results, the core target of cortex mori mainly involved nuclear receptor activity, ligand-activated transcription factor activity, steroid hormone receptor activity, RNA polymerase II-specific DNA-binding transcription factor, DNA-binding transcription factor, cytokine receptor, cytokine activity, etc. in the treatment of lung cancer (**Table 2**, **Figure 4A**). An enrichment analysis of KEGG pathways found 160 pathways associated with the treatment of lung cancer by cortex mori. It mainly included the diabetes complications mediated by the AGE-RAGE pathway, atherosclerosis, hepatitis B, prostatic carcinoma, Kaposi sarcoma-associated herpes virus infection, IL-17 signaling pathway, bladder cancer, TNF signaling pathway, small cell lung cancer, HIF-1 signaling pathway, non-small cell lung cancer, etc., as shown in **Table 3** and **Figures 4B**–**6** shows the signaling pathways involved with non-small cell lung cancer and small cell lung cancer.

**Table 2:** Partial results of GO enrichment analysis.

Order	Number	Biological function	P value	Number of genes
1	GO:0004879	Nuclear receptor activity	9.80E^-15^	12
2	GO:0098531	Ligand-activated transcription factor activity	9.80E^-15^	12
3	GO:0003707	Steroid hormone receptor activity	9.75E^-14^	12
4	GO:0061629	RNA polymerase II-specific DNA-binding transcription factor binding	1.06E^-11^	19
5	GO:0140297	DNA-binding transcription factor binding	1.09E^-11^	21
6	GO:0005126	Cytokine receptor binding	1.26E^-09^	17
7	GO:0005125	Cytokine activity	1.92E^-09^	15
8	GO:0001228	DNA-binding transcription activator activity, RNA polymerase II-specific	4.15E^-09^	20
9	GO:0001216	DNA-binding transcription activator activity	4.31E^-09^	20
10	GO:0044389	Ubiquitin-like protein ligase binding	2.67E^-08^	16
11	GO:0020037	Heme binding	4.83E^-08^	11
12	GO:0051879	Hsp90 protein binding	8.54E^-08^	7
13	GO:0046906	Tetrapyrrole binding	1.01E^-07^	11
14	GO:0030374	Nuclear receptor transcription coactivator activity	1.79E^-07^	8
15	GO:0005496	Steroid binding	2.28E^-07^	9
16	GO:0016705	Oxidoreductase activity	2.58E^-07^	11
17	GO:0019207	Kinase regulator activity	4.93E^-07^	12
18	GO:0031625	Ubiquitin protein ligase binding	5.01E^-07^	14
19	GO:0008227	G protein-coupled amine receptor activity	1.13E^-06^	7
20	GO:0097110	Scaffold protein binding	1.13E^-06^	7

**Table 3:** KEGG pathway enrichment analysis.

Order	Number	Biological function	P value	Number of genes
1	hsa04933	AGE-RAGE signaling pathway in diabetic complications	1.25E^-27^	29
2	hsa05418	Fluid shear stress and atherosclerosis	1.16E^-25^	31
3	hsa05161	Hepatitis B	2.58E^-22^	30
4	hsa05215	Prostate cancer	3.95E^-21^	24
5	hsa05167	Kaposi sarcoma-associated herpesvirus infection	4.08E^-21^	31
6	hsa04657	IL-17 signaling pathway	3.70E^-20^	23
7	hsa05219	Bladder cancer	1.33E^-19^	17
8	hsa04668	TNF signaling pathway	1.56E^-19^	24
9	hsa05163	Human cytomegalovirus infection	4.35E^-19^	31
10	hsa05212	Pancreatic cancer	2.52E^-18^	20
11	hsa05160	Hepatitis C	3.98E^-18^	26
12	hsa01522	Endocrine resistance	5.64E^-16^	20
13	hsa05142	Chagas disease	1.29E^-15^	20
14	hsa05222	Small cell lung cancer	2.51E^-15^	19
15	hsa04066	HIF-1 signaling pathway	5.00E^-15^	20
16	hsa05223	Non-small cell lung cancer	7.36E^-15^	17
17	hsa05169	Epstein-Barr virus infection	2.19E^-14^	25
18	hsa04625	C-type lectin receptor signaling pathway	2.74E^-14^	19
19	hsa05225	Hepatocellular carcinoma	2.94E^-14^	23
20	hsa05164	Influenza A	4.34E^-14^	23

### Molecular docking stimulation

**Figure 7:**
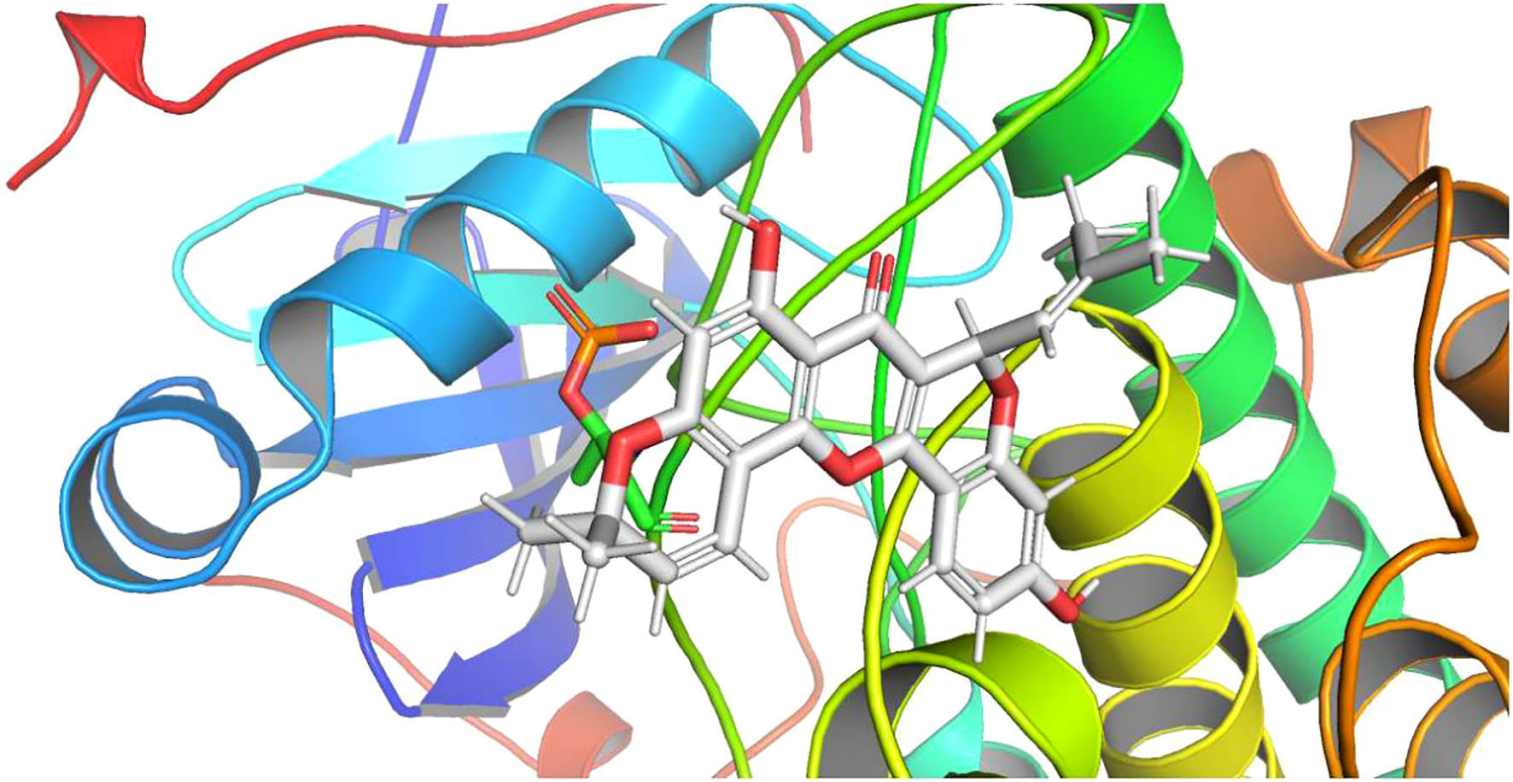
Molecular docking analysis of molecular interactions of AKT and the active ingredient cyclomorusin.

In drug design, molecular docking involves taking into account the characteristics of the receptor and the way it interacts with the drug molecule. A theoretical model that studies molecular interactions (e.g., ligands and receptors) and predicts binding patterns and affinity. Molecular docking has become an important technology in computer-assisted drug discovery in recent years (17, 18). Molecular covalent coupling occurs spontaneously when the binding energy is lower than 0, and smaller binding energies result in more stable conformations. In order to explore the possibility of interacting between the curative components within cyclomorusin and the target molecules (receptor = 6ccy_s, center_x = -9.801, center_y = 15.312, center_z = -31.398, size_x = 15, size_y = 15, size_z = 15, num_modes = 9), docking studies were implemented. **Figure 7** showed the binding mode with the best docking score. The molecular docking fraction value was -11.6. An increase in docking score is indicative of a greater affinity between the docking ligand and the protein receptor. With a lower docking score, a better affinity is reflected.

### The *in vitro* effects of cyclomorusin on lung cancer cells

**Figure 8:**
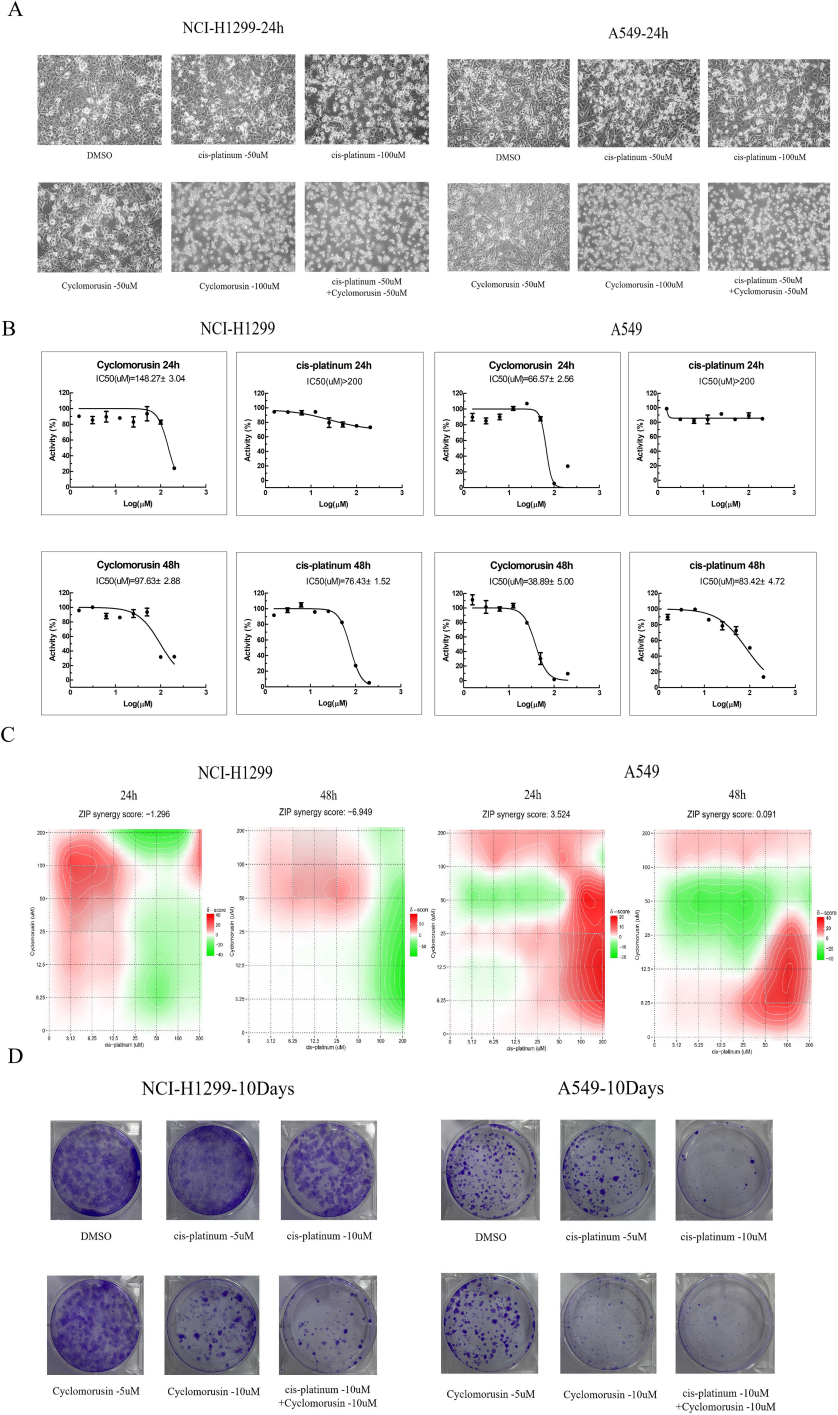
Inhibitive effect of Cyclomorusin on the growth of human lung cancer cells *in vitro*. **(A)** Lung cancer cells (NCI-H1299 and A549) were treated with 50 μM and 100 μM cyclomorusin for 24 h Cell morphological changes were determined under phase contrast microscope; **(B)** Lung cancer cells (NCI-H1299 and A549) were treated with a series of cyclomorusin and cis-platinum concentrations (0-200µM) for 24 h or 48 h followed by MTS assay; Values were mean ± SD (n = 3). **(C)** Lung cancer cells (NCI-H1299 and A549) were treated with a series of concentrations of cyclomorusin combined with cis-platinum (0-200 μM) for 24 or 48 h and then detected by MTS assay. ZIP synergy score was all greater than -10 and less than 10, indicating that cyclomorusin and cis-platinum had additive effect; **(D)** Foci formation of NCI-H1299 and A549 cells was determined. After indicated treatments, lung cancer cells were trypsinized and plated in duplicates at low density. After 10 days, formed colonies were stained with crystal violet.

MTS assays were carried out on A549 and NCI-H1299 cell lines to assess cortex mori’s inhibiting effect on lung cancer cell growth. The effects of cyclomorusin, cortex mori, and cis-platinum on lung cancer cell lines were first determined using MTS assays. Cyclomorusin and cis-platinum inhibit lung cancer cell viability dose- and time-dependently (**Figure 8B**). The IC50 value of cyclomorusin was lower than the IC50 value of cis-platinum in lung cancer cells for 24h, this results indicated that cyclomorusin might have better inhibitory effect than cis-platinum. Furthermore, after treatment with 50 and 100 µM cyclomorusin for 24 h, significant apoptotic morphological changes (such as smaller, round, and blunt in size) of lung cancer cells (NCI-H1299, and A549) were observed in a dose dependent manner compared to control groups (**Figure 8A**). The development of complex diseases such as cancer was often accompanied by changes in complex gene pathways, so drug combinations played a better therapeutic effect by acting on multiple pathways and multiple targets. Combined with cis-platinum, cyclomorusin and cis-platinum significantly reduced lung cancer cell viability, and their effects were additive (**Figure 8C**). Moreover, cyclomorusin could also suppress colony formation, demonstrating that cyclomorusin inhibited long-term survival of lung cancer cells (**Figure 8D**).

### PI3K-AKT pathway plays a pivotal role in cyclomorusin-induced lung cancer cells apoptosis

**Figure 9:**
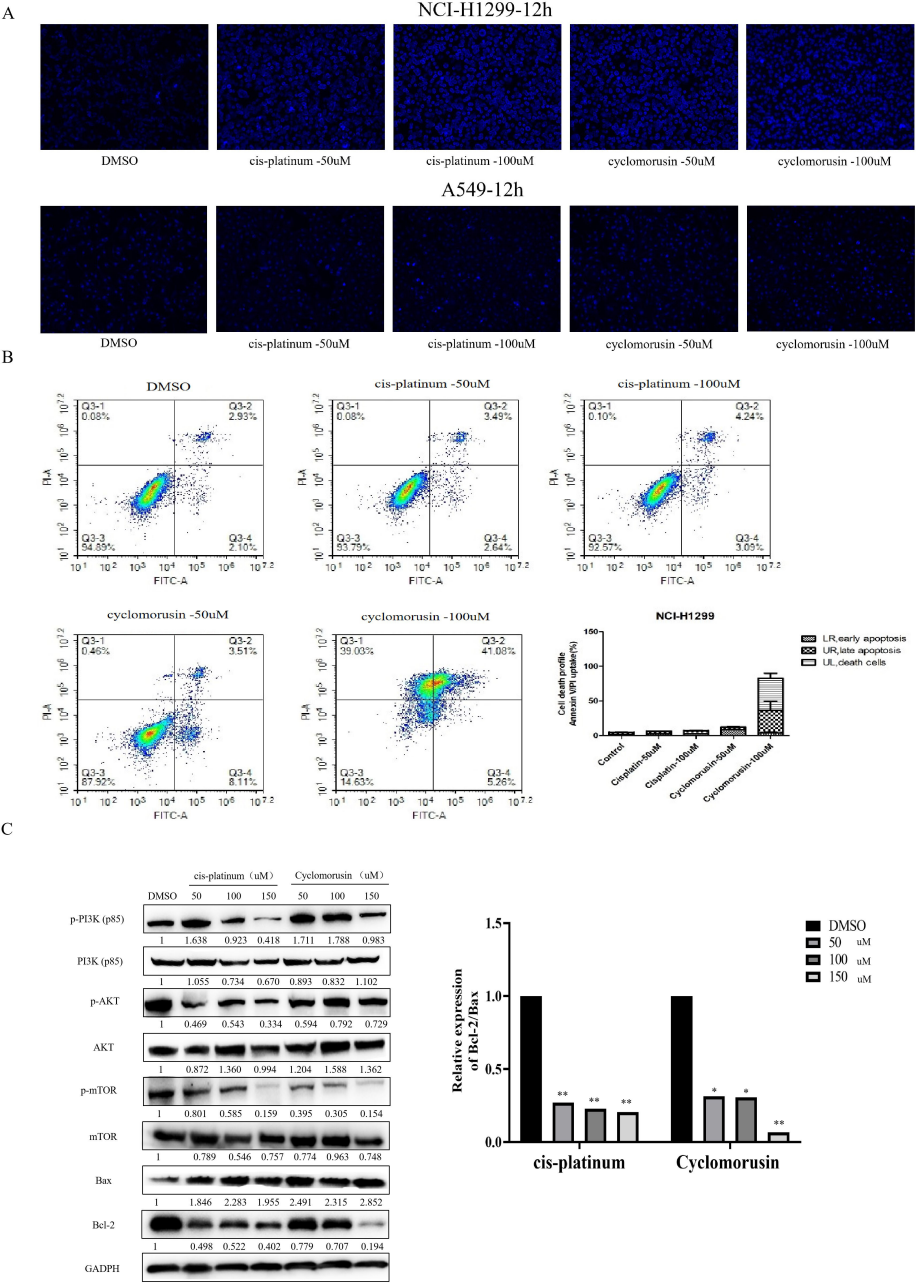
Effect of cyclomorusin on lung cancer cells death by restraining PI3K-AKT pathway. **(A)** Cells stained with Hoechst 33342 were detected and calculated by fluorescent photomicrographs; **(B)** A flow cytometric analysis of lung cancer cells (NCI-H1299) treated with different treatments was conducted using AnnexinV/PI. A cell death was observed in the right upper quadrant, as well as in the left upper quadrant. The values were the mean + SD (n=3); **(C)** After lung cancer cells were treated with cyclomorusin and cis-platinum for 24 h, protein levels of Bax, Bcl-2, PI3K, AKT, mTOR, p-AKT, p-PI3K, and p-mTOR were analyzed by western blot (*p < 0.05, **p < 0.01).

In order to further investigate the effect of cyclomorusin and cis-platinum on the apoptosis of lung cancer cells, Hoechst 33342 staining and Annexin V/Propidium Iodide (AnnexinV/PI) flow-cytometry assay were applied to measure cell apoptosis after treatment with cyclomorusin and cis-platinum. Hoechst 33342 staining showed that cyclomorusin caused condensed and/or fragmented nuclei in lung cancer cells (**Figure 9A**). And after 24 hours of treatment with cyclomorusin and cis-platinum, apoptosis in cells was measured using Annexin V/Propidium Iodide (AnnexinV/PI) flow-cytometry. As shown in **Figure 9B**, cyclomorusin and cis-platinum increased apoptosis of lung cancer cells in a dose-dependent manner. Cells treated with 100 μM cyclomorusin demonstrated a higher apoptosis rate than cells treated with 100 μM cis-platinum. An analysis of network pharmacology found that 160 pathways were related to the treatment of lung cancer by cortex mori, and including the PI3K-AKT signaling pathway, and PI3K-AKT signaling pathway is decisive to the occurrence and development of lung cancer cells. Western blot analysis showed that cyclomorusin and cis-platinum inhibited AKT phosphorylation, mTOR phosphorylation and PI3K (p85) phosphorylation, and decreased the ratio of Bcl-2/Bax in a dose-dependent manner (**Figure 9C**). In conclusion, these results manifested that PI3K-AKT was crucial for lung cancer cell apoptosis induced by cyclomorusin and cis-platinum.

### Detection of the effect of cyclomorusin on cell cycle and migration of lung cancer cells *in vitro*

**Figure 10:**
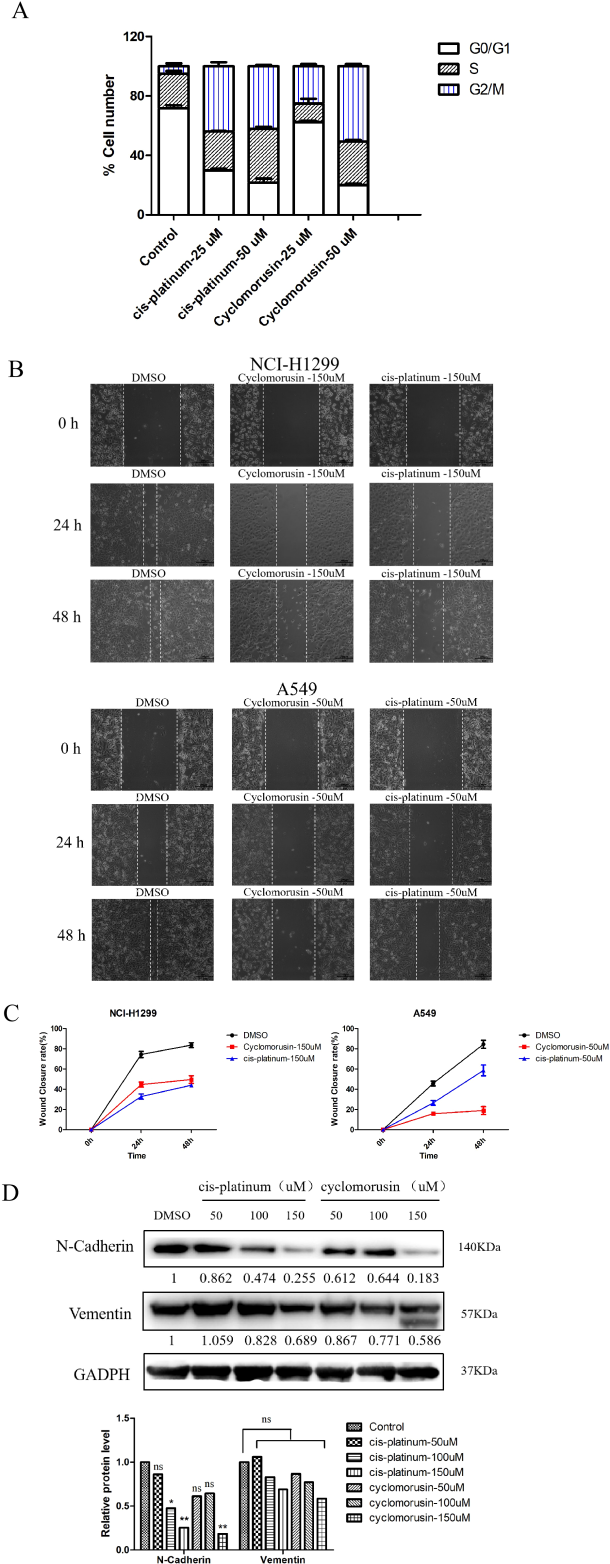
Metastasis and cell cycle inhibition by cyclomorusin in lung cancer cells. **(A)** The effect of cyclomorusin at various levels on the advancement of the cell cycle was examined using propidium iodide flow cytometry after 24 h; **(B)** Inhibitive effects of cyclomorusin and cis-platinum on lung cancer cell migratory ability. The smaller the scratch, the stronger the migration ability; **(C)** Wound closure rate of the lung cancer cells with different treatments. **(D)** The effect of cyclomorusin and cis-platinum on proteins related to metastasis. (p = ns (no significance), *p < 0.05, **p < 0.01).

After receiving a signal to induce apoptosis, the cell cycle can be changed, such as the activation of cyclin or the arrest of the cell cycle. Moreover, in many systems, apoptosis can be accelerated through the arrest of the cell cycle. Flow cytometry was used to analyze lung cancer cells’ cycle following exposure to cyclomorusin and cis-platinum for 24 h. Compared with the control, cyclomorusin significantly arrested the cell cycle in the G2/M phase, as shown in **Figure 10A** and **Supplementary Table S1**. The proportion of G2/M phase cells increased from 4.90 ± 1.90% (control group) to 50.63 ± 1.42% (cyclomorusin 50μM). Meanwhile, there was a reduction in the percentage of the cells in the G0/G1 phase (control: 71.61 ± 2.08%, cyclomorusin, 50 μM: 19.93 ± 1.16%). In summary, cyclomorusin induced the cell cycle to stop in G2/M phase, leading to the gradual initiation of apoptosis in the cells.

The metastasis of cancer remains one of the most important challenges in cancer treatment. Therefore, inhibiting migration may be an effective cancer treatment strategy. To validate the effect of cyclomorusin on tumor cell migration, wound healing assays were performed. In order to confirm the inhibition effect of cyclomorusin in lung cancer metastasis, lung cancer cells were treated with cyclomorusin and cis-platinum for 24 and 48 hours, respectively. At 24 hours and 48 hours, vehicle-treated lung cancer cells covered more than 80% of the gap in wound healing assays. In contrast, lung cancer cells treated with cyclomorusin or cis-platinum were mostly uncovered at 24 hours and 48 hours (**Figures 10B, C**). The western blot analysis showed that cyclomorusin and cis-platinum decreased the protein (N-Cadherin and Vementin) expression of markers in NCI-H1299 cells as well as their migration ability in a dose-dependent manner. According to **Figure 10D**, 150μM cyclomorusin has a superior inhibitory effect on N-Cadherin and Vementin when compared to cis-platinum.

### The effects of cyclomorusin on zebrafish embryo development

**Figure 11:**
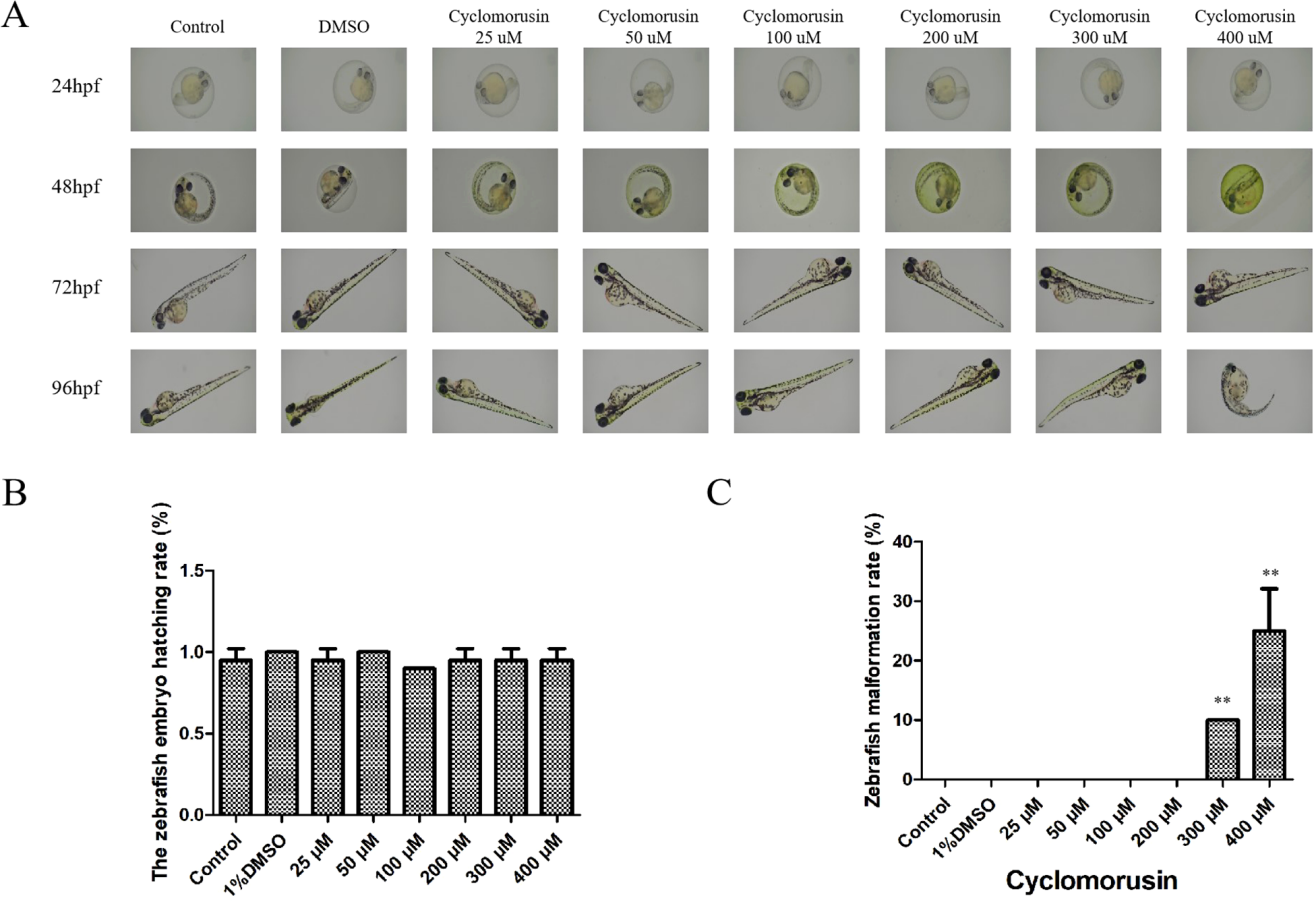
The effect of cyclomorusin on zebrafish embryo development. **(A)** The growth of zebrafish embryos was observed at 24, 48, 72, and 96 h after various treatments using a microscope; **(B)** The zebrafish embryo hatching rate; **(C)** The zebrafish malformation rate (**p < 0.01).

The use of zebrafish embryos to screen compounds *in vivo* has become increasingly popular and has been used to test the effectiveness and harmfulness of drugs. The occurrence of toxicity in zebrafish embryos is related to teratogenesis and mortality. After exposure to cyclomorusin for 24, 48, 72, and 96 h, the survival and early development of zebrafish embryos were observed by light microscopy (**Figure 11A**). Embryos incubated with 0.1% DMSO showed no significant differences compared to untreated embryos. During the observation period, the untreated embryos did not have any death or developmental malformations. Interestingly, zebrafish embryos were well tolerated by cyclomorusin at the effective dose (200 μM) and had no significant toxicity. However, the zebrafish embryo hatching rate exposed to cyclomorusin had no significant toxicity at 96h post-exposure (**Figure 11B**). Developmental abnormalities such as hemorrhages, oedemas, tail scoliosis, or delayed hatching were observed when zebrafish were implanted with cyclomorusin (400μM), whereas the incidence of these abnormalities was lower at 300 μM doses, it was only 10% at 96h post-exposure (**Figure 11C**). To sum up, we have reason to believe that the relative safe concentration of cyclomorusin for zebrafish embryos is < 200μM.

### *In vivo* anti-tumor assays

**Figure 12:**
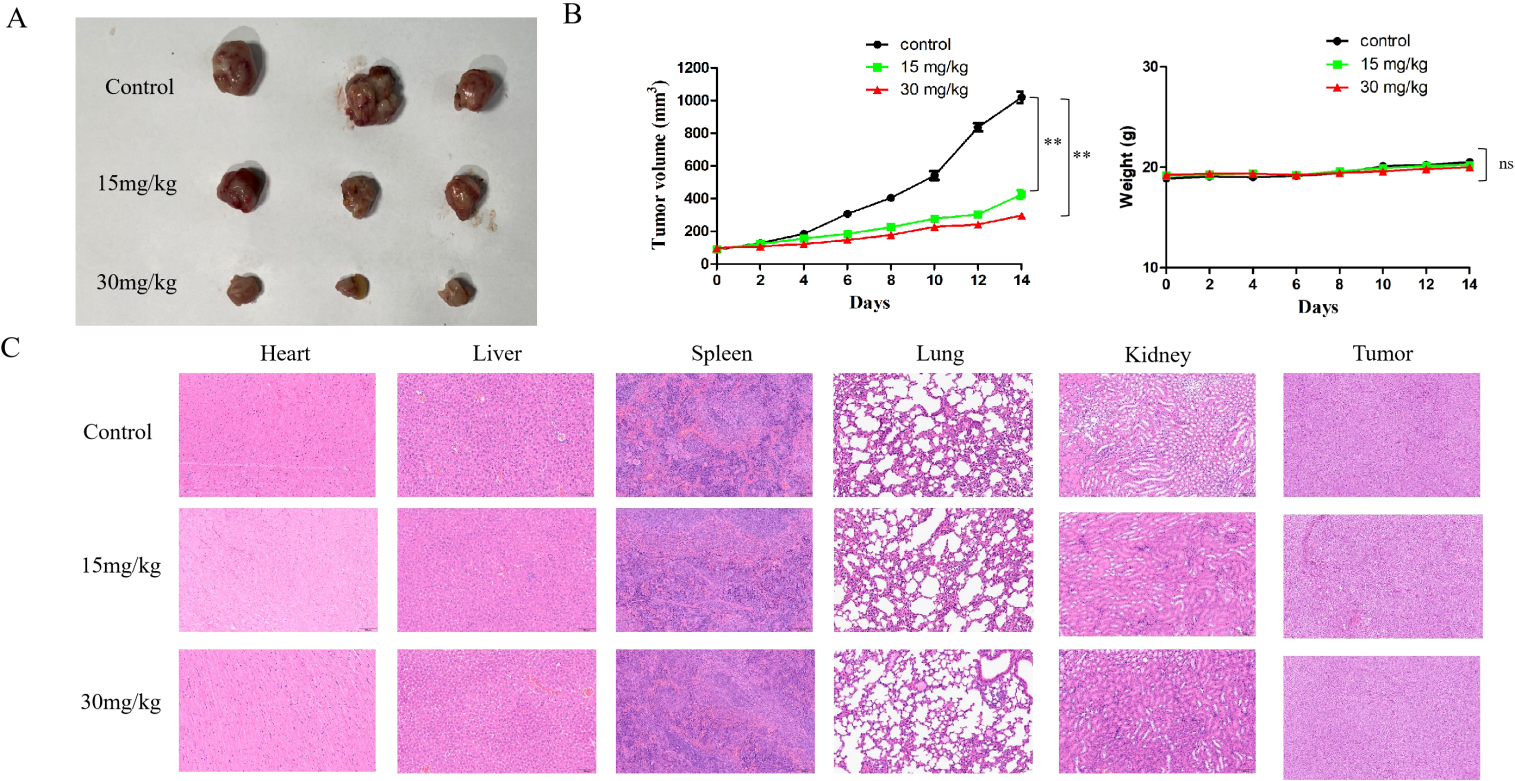
The *in vivo* experiments of cyclomorusin. **(A)** Tumor images of the control group and the experimental group receiving cyclomorusin treatment; **(B)** The effect of cyclomorusin on tumor volume after resection and on mice body weight (p = ns (no significance), **p < 0.01); **(C)** Illustrative images of H&E staining of the heart, liver, spleen, lung, kidney, and tumor.

Finally, a mouse model was used to examine if cyclomorusin hindered the tumor proliferation *in vivo*. At first, three groups of mice were implanted with lung cancer cells. Following the administration of cyclomorusin, observations were conducted every two days to monitor tumor growth and changes in the body weight of the mice. Compared with the normal saline control group, both cyclomorusin (15 mg/kg) and cyclomorusin (30 mg/kg) treatment resulted in reduced tumor volume, and the tumor inhibition was more significant in the cyclomorusin (30 mg/kg) group (**Figure 12A**). In addition, the t-test results showed significant differences between the two interventions. To be note, there was no significant weight loss in the mice in all groups (**Figure 12B**). An organslicing procedure was performed after 15 days on all mice. H&E staining results showed no indications of damage to the heart, liver, spleen, lungs, and kidneys in all mice after cyclomorusin treatment (**Figure 12C**). In summary, these *in vivo* studies showed that cyclomorusin had high anti-cancer efficacy and minimal side effects.

## Discussion

Our study examined the pharmacological effects of cortex mori medicinal ingredients in lung cancer disease targets via network analysis. Cyclomorusin, kuwanon D, Moracin A and moracin O are identified as the active ingredients related to the most targets. The drug-likeness value of cyclomorusin is the top 1 among the major active ingredients in cortex mori. In addition, docking studies showed that cyclomorusin has a good affinity for the target protein AKT1. In nature, cyclomorusin is a common flavonoid compound. Many studies have shown that most of the flavonoids compound in traditional Chinese medicine have strong antioxidant activity (19–21). Cyclomorusin has been confirmed to have the dual effect of inhibiting and promoting uric acid excretion (22). Until present, there is no relevant literature on cyclomorusin in lung cancer cells. Therefore, this experiment provides a good reference value for the future study of cyclomorusin in more tumor cells. Traditional Chinese medicine has the characteristic of less side effects, which provides a new treatment strategy for improving the side effects of chemotherapy drugs in clinical practice (21). However, the specific ingredients of traditional Chinese medicine are difficult to identify, and the specific mechanism is also difficult to explain clearly. This study investigated the mechanism of action of cortex mori in treating lung cancer using a network pharmacology approach, and the purpose of this study was to provide a new method and direction for the development of traditional Chinese medicine of cortex mori.

By the network pharmacological analysis, 32 active ingredients were performed, such as cyclomorusin, cyclocycin D, and cyclocycin A were screened out, and 434 traditional Chinese medicine targets were obtained. According to the traditional Chinese drug-disease target protein interaction network and core target bar chart, JUN, AKT1, MAPK1, RELA, IL6, MAPK14, MAPK8, ESR1, FOS, CXCL8 and EGFR might be the core genes that exert anti-lung cancer effect of cortex mori. In tumorigenesis, AKT subtype AKT1 has dual functions, acting both as an oncogene inhibiting apoptosis and an anticancer gene inhibiting invasion and metastasis, which plays a dual role (23). Lung cancer differentiation, lymph node metastasis, and stage are closely associated with AKT1 expression (24). In our study, we studied that the PI3K-AKT pathway plays a key role in cyclomorusin-induced apoptosis in lung cancer cells, but the role of JUN in cyclomorusin-induced apoptosis in lung cancer cells need to be further explored. This study enriched 160 pathways in total, including IL-17 signaling pathway, TNF signaling pathway, small cell lung cancer, HIF-1 signaling pathway, non-small cell lung cancer and so on. In small cell lung cancer and non-small cell lung cancer pathways, the PI3K-AKT signaling pathway and p53 signaling pathway can inhibit the cell cycle and cell apoptosis of lung cancer. A number of studies have been conducted on the PI3K-AKT-mTOR signaling pathway, which is an important pathway in signal transduction in cells, and it is closely related to the occurrence and development of lung cancer by influencing the activation state of various effector molecules downstream (25, 26). In addition, KEGG pathway enrichment analysis reveald that in addition to anti-inflammatory effects, cortex mori also had certain antiviral activities, which also suggested that cortex mori might have certain adjuvant therapeutic effects in the outbreak of COVID-19.

Cell biology experiments *in vitro* showed that cyclomorusin, the active component of cortex mori, had a certain inhibitory effect on lung cancer cells in a time- and dose- concentration dependent manner and had additive effect with cisplatin against lung cancer cells. When treated lung cancer cells with cyclomorusin or cisplatin for 24h, the results of flow cytometry analysis revealed that cyclomorusin promoted the apoptosis of lung cancer cells significantly better than cisplatin. Relevant studies have confirmed that inhibition or reduction of phosphorylation of PI3K/AKT signaling pathway related proteins can inhibit the occurrence and development of tumor cells (27–29). A number of studies have demonstrated abnormal activation of PI3K/AKT signaling in malignant tumors, which may have a bearing on the occurrence and development of tumors, regulates cell proliferation, survival, transformation, adhesion and extracellular matrix degradation, and participates in the whole process of tumors, such as liver cancer, pancreatic cancer and gastrointestinal tumors (30–32). Drug resistance in tumors is also related to this pathway, according to studies. Zhang JY et al. pointed out that PI3K-AKT signaling pathway may increase the resistance to 5-FU by promoting the expression of P-glycoprotein in human colorectal cancer HCT-8/FU-resistant cells (33).

According to our western blot results, cyclomorusin could decrease PI3K and AKT phosphorylation, decrease the ratio expression of BCL-2/Bax, and decrease the expression of migratory protein N-Cadherin and Vementin. In summary, cortex mori can act on lung cancer through cyclomorusin, morus flavone D, and sanxin A, which reflects the characteristics of multi-target and multi-pathway treatment of lung cancer, and provides theoretical basis for the clinical application and experimental research of cortex mori, and provides a new direction for the treatment of lung cancer. In this study, network pharmacological analysis combined with cell biology experiment were used to explore the effect of cortex mori and cyclomorusin on the treatment of lung cancer, which made the results of network pharmacological analysis more reliable and fully confirmed the effect of cyclomorusin on lung cancer. Furthermore, it allows traditional Chinese medicine to be applied in a wider range of clinical settings in the future. However, network pharmacology has deficiencies in aspects such as database quality, prediction accuracy, standardization, resource requirements and individual differences. These problems need to be gradually solved through further technological innovation, data accumulation and interdisciplinary cooperation (34). In addition, the differences between experimental models and clinical reality also need to be considered in the future medical application of cyclomorusin.

## Conclusion

In our study, cyclomorusin represents a potential effective chemical compound for treating lung cancer and can provide insights into the development of cortex mori in lung cancer treatment. In addition, the pharmacological effects of cortex mori and the major ingredients cyclomorusin were comprehensively discussed from both theoretical and practical aspects by network pharmacological analysis combined with cell biology experiments, which also offers a certain reference for the development of traditional Chinese medicine in the future. However, the limitations of network pharmacology prediction, the differences between experimental models and clinical reality, the small sample sizes *in vivo* and the potential off-target effects in our study need to be considered in the future medical application of cyclomorusin.

## Data Availability

The original contributions presented in the study are included in the article/**Supplementary Material**, further inquiries can be directed to the corresponding author/s.
