# Supplementary material for: Molecular targets and mechanisms of cortex mori for lung cancer treatment: a network pharmacology study, molecular docking and in vitro and in vivo experimental validation
Source: Front Oncol. 2025 May 29;15:1587856. doi: 10.3389/fonc.2025.1587856 (PMC12158998; doi:10.3389/fonc.2025.1587856)
Supplement: Supplementary file 1 [file Table1.docx]

Table S1 Effect of cis-platinum and Cyclomorusin on the distribution of NCI-H1299 cell in cell cycle analysis after 24 h treatment ^a^

| Complexes | G_0_/G_1_ | S | G_2_/M |
| --- | --- | --- | --- |
| Control | 71.61±2.08 | 23.49±1.78 | 4.90±1.90 |
| cis-platinum, 25 μM | 29.87±1.23*** | 26.24±0.33 | 43.89±2.75*** |
| cis-platinum, 50 μM | 21.68±2.90*** | 36.31±1.27*** | 42.01±0.94*** |
| Cyclomorusin, 25 μM | 62.36±1.02** | 12.58±3.25** | 25.06±1.41*** |
| Cyclomorusin, 50 μM | 19.93±1.16*** | 29.44±1.09** | 50.63±1.42*** |

^a^ Data are presented as mean values ± SD of three independent experiments for each treatment. (*p < 0.05，**p < 0.01，***p < 0.001)
